# Influence of Partially Substituting Wheat Flour with Tiger Nut Flour on the Physical Properties, Sensory Quality, and Consumer Acceptance of Tea, Sugar, and Butter Bread

**DOI:** 10.1155/2023/7892739

**Published:** 2023-01-17

**Authors:** Nazir Kizzie-Hayford, Joshua Akanson, Jerry Ampofo-Asiama, Ernest Ekow Abano

**Affiliations:** ^1^Department of Biochemistry, School of Biological Science, College of Agriculture and Natural Sciences, University of Cape Coast, Cape Coast, Ghana; ^2^Department of Agricultural Engineering, School of Agriculture, College of Agriculture and Natural Sciences, University of Cape Coast, Cape Coast, Ghana

## Abstract

Tiger nut is a valuable source of fiber, lipids, minerals, and carbohydrates. However, avenues for incorporating tiger nuts into food remain underexplored, especially in several tropical countries where the plant grows well. The current study investigated the effects of partially substituting wheat flour (WF) with tiger nut flour (TNF) on the physical and sensory properties of different bread types to evaluate the more amenable system for tiger nut incorporation. The substitution was done at WF:TNF ratio of 100 : 0, 90 : 10, 85 : 15, 80 : 20, 75 : 25, and 70 : 30 for butter bread (B_b_), tea bread (T_b_), and sugar bread (S_b_). The results show that WF substitution with TNF increased bread brownness and color saturation and decreased lightness, showing the highest impact on S_b_, followed by T_b_ and B_b_. Additionally, bread-specific volume decreased significantly after 20% (B_b_), 25% (T_b_), and 30% (S_b_) TNF substitution. Furthermore, substituting WF with 30% TNF increased crumb hardness from approx. 1.87 N to 3.64 N (B_b_), 3.46 N to 8.14 N (T_b_), and 6.71 N to 11.39 N (S_b_) and caused significant increases to 17.80 N (T_b_) and 21.08 N (S_b_) after 3 d storage. Only a marginal effect on storage hardness (4.32 N) was observed for B_b_. Substituting WF with 10% TNF for B_b_ or 25% TNF for T_b_ led to significantly higher consumer (*N* = 56) scores for all attributes and overall acceptability. However, no significant effect on the overall acceptability of S_b_ was observed. Flash profiling showed frequently used descriptors for B_b_ as *firm*, *moist*, *buttery*, *smooth*, and *astringent.* After 10% TNF substitution, descriptors were *chewy*, *firm*, *sweet*, *porous*, *dry*, and *caramel*, and that of 30% TNF were *grainy*, *chocolate*, *brown*, *nutty*, and *flaky*. Substituting WF with TNF increased the lipids, fiber, and minerals content but decreased the protein and carbohydrate contents of bread. TNF substitution led to different physical and sensory effects depending on bread type, showing that B_b_ with 10% or T_b_ with 25% TNF is more comparable with the overall acceptance quality of 100% WF. The study is relevant for utilizing tiger nuts as an ingredient in bread products.

## 1. Introduction

Tiger nut (*Cyperus esculentus* L) is a tuberous rhizome belonging to the Cyperaceae family of grass-like plants. The edible, almond-like tuber grows in temperate and several tropical regions and is rich in fiber and lipids comprising high content of mono- and polyunsaturated fatty acids, carbohydrates, and some proteins. The nut-like tuber is also rich in minerals, vitamins C and E, and several bioactive phytochemical compounds [1, 2], showing that tiger nuts can be a valuable source of food. Recently, interest in processing tiger nut has revealed several possibilities such as the aqueous extraction of tiger nut into vegetable milk derivatives [3–5] and the milling of tiger nut into flour for biscuits [6] and bread [7], egg tagliatelle [8], pasta, and other related extruded products [9, 10]. Thus, the exploration of tiger nut usage remains relevant for creating tasty and nutritious foods, as well as improving the technofunctional properties of existing foods.

Indeed, the incorporation of tiger nut in the formulation of bread, which is a universal ready-to-eat worldwide staple [11], has received additional attention because of the simplicity of the production process and ingredient availability and flexibility of incorporating different flour types [12]. The use of tiger nut in bread baking can lead to possibilities for creating healthier products with enhanced minerals and fiber [6] whilst serving as an avenue for utilizing the underexplored, less known, nutrient-rich tuberous rhizome for food [13].

A few reported studies showed that partially substituting wheat flour with tiger flour by more than 8% significantly reduced bread-specific volume, crumb moisture, and dough extensibility, resulting in decreased consumer acceptance [14]. Others showed that partial substitution of wheat flour bread allowed up to 15% tiger nut flour without detracting significantly from the sensory properties [13]. Whilst the method of preparation is likely to influence the use of tiger nut flour in bread making, the type of bread under consideration could influence the allowable levels of flour substitution and consumer acceptability. Limited information exists on the effect of partial wheat flour substitution with tiger nut flour on the physical and sensory properties of different bread types even though distinct effects may arise, which could create possibilities for improving bread functionality and/or enhancing the utility of tiger nut in bread processing. Taking into account that bread making is an art, and that, several formulations and recipes exist to meet the unique regional, continental, cultural, and nutritional needs of consumers [12], assessing the effect of partially substituting wheat flour with tiger nut flour on the quality and acceptability of different bread types is fundamental. In many African countries such as Ghana, Nigeria, and Kenya, several bread types with unique attributes are consumed including sugar bread, butter bread, tea bread, rye bread, whole wheat bread, and sourdough bread to mention a few [15]. For a case study, we selected three commonly consumed bread types in Ghana, namely, butter bread, tea bread, and sugar bread [15]. Then, the suitability of partially substituting wheat flour with tiger nut flour was tested to understand the impact on the physical properties and bread attributes and to know the bred type that is more amenable to the process as well as the levels of substitution that promises the highest consumer acceptability. This study is crucial for designing a process for tiger nut utilization in bread processing, which could be a useful avenue for integrating tiger nuts into the food chain.

## 2. Materials and Methods

Freshly harvested tiger nut was obtained from local farmers in Twifo Praso in the Central Region, Ghana. Hard wheat flour (WF), (Pride of the West) supplied by Iran brothers and others Ltd., Ghana, was obtained from the Kotokoraba Supermarket, Cape Coast, Ghana. Granulated sugar, commercially available bakery shortening (margarine), yeast, nutmeg, and salt commonly used for baking were obtained from the Kotokoraba Supermarket trading center at Cape Coast, Ghana.

### 2.1. Preparation of Tiger Nut Flour

Tiger nuts were hand-picked to exclude discolored or broken pieces and stones and washed with water to eliminate dirt, adhering root hairs, and soil [4]. To enhance drying efficiency, tiger nuts were pulverized using a cutting blender (Ninja, 110 V, 1001 W, Needham, Massachusetts, USA) and dried in an oven (Memmert UM-400, Germany) at 70°C for 13 h before milling into a fine powder using a locally fabricated hammer mill (Intermediate Technology Transfer Unit, Cape Coast, Ghana) to pass a 0.3 mm sieve to obtain the tiger nut flour (TNF) as previously reported [16]. Then, TNF was transferred into zip-lock bags and kept in a refrigerator at 4°C until use.

### 2.2. Preparation of Bread and Analogs with Tiger Nut Flour

For making tiger nut-substituted bread, a Minitab software was used for a centroid mixture design whereby WF was substituted with TNF for butter bread (B_b_), tea bread (T_b_), and sugar bread (S_b_) at five (5) levels, namely, 10%, 15%, 20%, 25%, or 30%. Then, the procedure for bread preparation reported by Aryeetey et al. [15] was adopted with a few modifications: the appropriate mass of ingredients was weighed according to Table 1. Salt and sugar were dissolved in a measured portion of water, and the yeast was added for preconditioning at ambient temperature (28°C-32°C) for 60 min. Flour, nutmeg, and margarine were separately weighed and hand-mixed to obtain evenly mixed ingredients. After adding the yeast solution to the flour, the mixture was rubbed together to obtain a rough dough followed by kneading using a rolling machine to obtain a fine-textured dough [13]. Based on the recipe in Table 1, WF substitutions resulted in B_b_ and S_b_ with TNF compositions of 7.7%, 11.6%, 15.4%, 19.3%, and 23.2% and for T_b_, 8.5%, 12.7%, 17.0%, 21.2%, and 25.4%. Bread prepared using only WF served as the control. For each type of bread, 1000 g dough was sliced, rolled, molded, and arranged in a baking pan and covered with a clean cloth in an enclosed room to allow for proofing at ambient temperature (28-32°C) for 60 min. Then, the dough was transferred to a Swiss baking oven maintained at 230°C for 30 min [13]. The baked bread was transferred to a cooling room to equilibrate at room temperature and packed in translucent polythene bags until analyses. Baking experiments were done in duplicate.

## 3. Physical Analyses of Bread

### 3.1. Color of Crust and Crumb

Color properties of the bread crust and the crumb were measured based on the Hunter parameters (*L*^∗^, *a*^∗^, and *b*^∗^) using a hand-held portable colorimeter (CHN Spec, CS-10, Baoshishan, China). The instrument was calibrated against the white and black tiles, and the mean values for lightness (*L*^∗^), red–green intensity (*a*^∗^), and yellow–blue intensity (*b*^∗^) were calculated from the color primaries. Chroma was computed using equation (1) and used as an indicator of color saturation [17]. Browning index (BI), which is the measure of brown color purity, was also calculated using equation (2) according to Dabels et al. [18].

(1) $$\begin{array}{c} \text{Chroma }C^{*}={\left[\left(a^{*2}\right)+\left(b^{*2}\right)\right]}^{1/2}, \end{array}$$

(2) $$\begin{array}{c} \text{Browning index }\left(\text{BI}\right)=\frac{100\left(x-0.31\right)}{0.17},x=\frac{a^{*}+1.75L}{5.645L+\text{a}-3.01b^{*}}. \end{array}$$

### 3.2. Specific Volume

The specific volume of bread was determined by measuring the weight and the volume by the displacement method according to the procedure by Ahemen et al. [11] with a few modifications: after displacement, the volume of the residual millet grains was measured by transferring into a graduated cylinder and recorded in cm^3^ [13]. Specific volume was computed according to Dabels et al. [18] using equation (3) as follows:

(3) $$\begin{array}{c} \text{SV}\left(\frac{{\text{cm}}^{3}}{g}\right)=\frac{v}{w}, \end{array}$$

where *v* (cm^3^) is the volume and *w* (g) is the weight of the bread.

### 3.3. Crumb Hardness during Storage

Surface firmness of bread crumb was measured on the first day of baking and at 24 h intervals for 3 d using a modified penetrometer (Model GY 4 Fuzhou, Hedao) as described by Manohar and Rao [19] with slight modifications: bread was sliced to 5 cm thickness, and a 35 g conical probe was allowed to fall under gravity unto bread crumb from the hanger position. Cone compression time along the diagonal line of the breadcrumb was 3 s, and the depth of penetration (N) was recorded from the radial gauge and used as the measure of firmness. The arithmetic mean of results related to the physical properties of bread is based on triplicate experiments.

### 3.4. Sensory Analyses

Consumer acceptability was determined by using a 9-point hedonic scale as described by Lawless and Heymann [20]. Attributes related to crust and crumb appearance, aroma, mouthfeel, aftertaste, and overall acceptability were rated from 9-like extremely to 1-dislike extremely. Samples of bread analyzed were T_b_, B_b_, and S_b_. Each type of bread had five WF:TNF substitution levels, namely, 90 : 10, 85 : 15, 80 : 20, 75 : 25, and 70 : 30. Bread prepared with only WF (100 : 0) served as the control. About 30 g samples were given random 3-digit codes and served to a randomly recruited 56-member panel (males, 42; females, 14; average age, 23 years) in a counterbalanced manner.

Based on the results of the hedonic study, the bread that showed the highest overall consumer acceptance (B_b_ with TNF of 10%) and its analogs was chosen and flash-profiled [21] to determine the influence of TNF substitution on bread attributes.

A panel of 12 members (males, 5; females, 7; average age, 22 y) was randomly recruited and presented B_b_ with WF:TNF substitution ratios as follows: 100 : 0 (B_b_), 90 : 10 (T_10_B_b_), 85 : 15 (T_15_B_b_), 80 : 20 (T_20_B_b_), 75 : 25 (T_25_B_b_), and 70 : 30 (T_30_B_b_). An identical sample from T_20_B_b_ was included to assess the discriminatory quality. Samples were given 3-digit random codes and served in small trays. A bottle of water (300 mL) was added to rinse the mouth between samples. Raw data on the attributes and the corresponding intensities were analyzed by principal component and Generalized Procrustes Analyses using the Senstools. Net software (OP&P Product Research BV, Utrecht, Netherlands). Results on hedonic assessment and flash profiling of bread attributes are based on single and duplicate experiments, respectively.

### 3.5. Compositional Analyses

Compositional analyses of WF bread and the TNF-substituted analogs that showed the highest consumer acceptance (Table 2), namely, butter bread with 10% tiger nut flour (T_10_B_b_), tea bread with 25% tiger nut flour (T_25_T_b_), and sugar bread with 30% tiger nut flour (T_30_S_b_), were carried out. Additionally, the composition of TNF was analyzed to help determine its contribution to the different bread samples. Proximate analysis was carried out as explained previously [4]. Briefly, moisture content was determined by gravimetry using a forced draft oven. Protein was determined based on free nitrogen analyses (*N* × 6.25) using the Kjeldahl method. Fat was determined by acid digestion followed by Soxhlet extraction with diethyl ether. Total fiber content was analyzed using the sequential acid and alkaline digestion method. Ash content was analyzed by combusting in a muffle furnace, and carbohydrates were determined by difference [22]. Results are based on the arithmetic mean of triplicate experiments.

### 3.6. Statistical Analyses

To determine the effects of partially substituting wheat flour with tiger nut flour at a ratio of 100 : 0, 90 : 10, 85 : 15, 80 : 20, 75 : 25, and 70 : 30 for butter bread, tea bread, and sugar bread on the response parameters, a Minitab software was used. Data were evaluated using a one-way analysis of variance. Tukey HSD or Games-Howell post hoc analysis was used to compare the mean values where it is necessary. SPSS software package version 16.0 was used for performing the analysis (SPSS Inc., Chicago, IL, USA). All significance statements refer to *p* < 0.05.

## 4. Results and Discussion

### 4.1. Effects on Color

**Figure 1:**
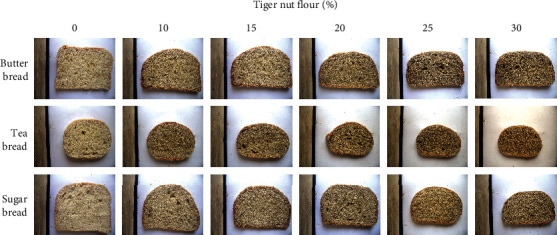
Cross-sections of butter bread, tea bread, and sugar bread produced by partial substitution of wheat flour with tiger nut flour. Numbers refer to percentage tiger nut flour substitution.

**Figure 2:**
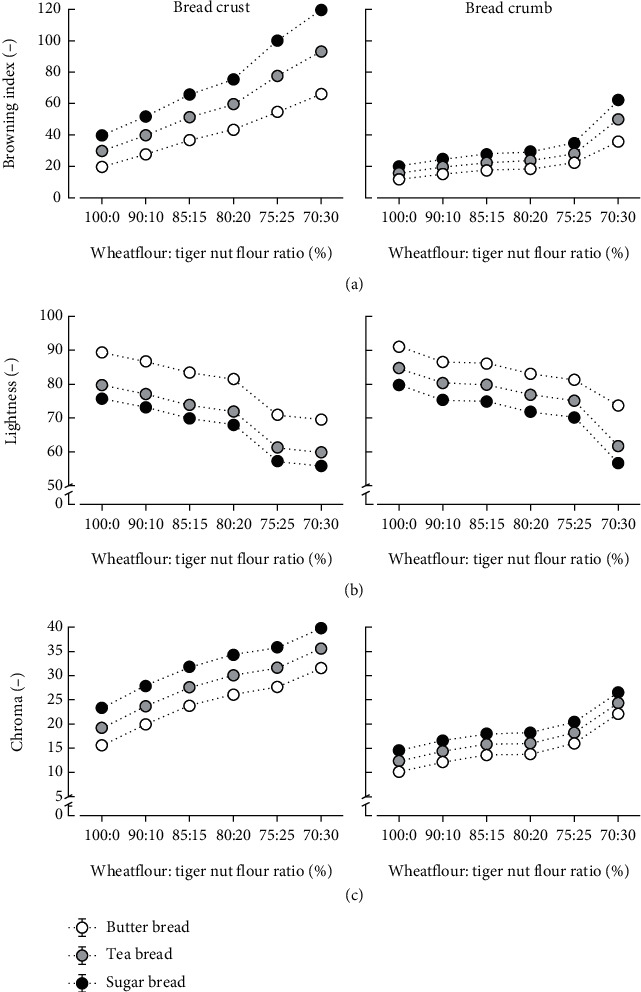
Effects of substituting wheat flour with tiger nut flour on browning index (a), lightness (b), and on Chroma (c) of the crust and crumb of butter bread, tea bread, and sugar bread.

Figures 1 and 2(a) show a significantly higher browning index (BI) of crust than crumb for all types of bread, which is consistent with the characteristics of most commercial bread.  Figure 2(a) shows that the crust and crumb of sugar bread (S_b_) gave the highest browning index (BI) followed by that of tea bread (T_b_) and butter bread (B_b_). Different browning intensities of bread could be caused by the variations in sugar composition, which is known to caramelize and also react with amino acids in a Maillard process during baking, contributing golden brown color compounds to the product [23]. Substituting WF with TNF progressively increased the browning index of the bread, giving a near-linear relationship with a correlation, *r* = 0.995 (B_b_), *r* = 0.992 (T_b_), and *r* = 0.989 (S_b,_), and a gradient or magnitude (BI/Unit) of 9.2 ± 0.04 (B_b_), 12.5 ± 0.06 (T_b_), and 15.8 ± 0.05 (S_b_) for the bread crust, showing that tiger nut addition significantly impacts crust of S_b_ the most, followed by T_b_ and B_b_. The crumb showed lower browning effects compared to the crust, and after adding 25% TNF, a more pronounced effect on the crumb brownness was observed. The results agree with the report according to Oke et al. [14], which indicated that adding TNF to bread increased browning. Tiger nut flour shows a deep brown color, which is a contribution from the outer color and the formation of caramel and Maillard products from the tiger nut drying process [16]. Here, we additionally show that substituting WF with TNF causes different intensities of browning depending on the type of bread, which can influence consumer acceptability.

Color primaries (lightness, *L*^∗^) and Chroma of the bread crust and crumb in Figures 2(b) and 2(c), respectively, corroborate the browning effects of TNF substitution by showing lower values of *L*^∗^ and higher values for color saturation (Chroma) when compared to the other bread types without TNF. Crust and crumb lightness of B_b_ was comparable, whilst significantly lower values of crust lightness than crumb lightness were observed for T_b_ and S_b_. TNF addition diminished *L*^∗^, showing more pronounced effects on the crust or the crumb after 20% TNF or 25% TNF addition, respectively. Additionally, TNF improved the redness (*a*^∗^) and yellowness (*b*^∗^) of bread, causing a more drastic effect on the color saturation of the crust than the crumb after 25% TNF addition. The effects of TNF substitution on the crust and crumb appearance could be relevant for modulating bread color properties. Browning and other color attributes play a major role in the consumer acceptability of bread, besides the contribution to flavour and aroma. However, the browning reactions can adversely affect nutritional value due to amino acid-simple sugar Maillard-type reactions and the possible formation of acrylamide-related compounds [23]. The browning effects of tiger nuts could be a useful factor that could be exploited for modulating bread color, which could be useful for determining the bread type that is more amenable to tiger nut flour incorporation.

### 4.2. Effects on Specific Volume

**Figure 3:**
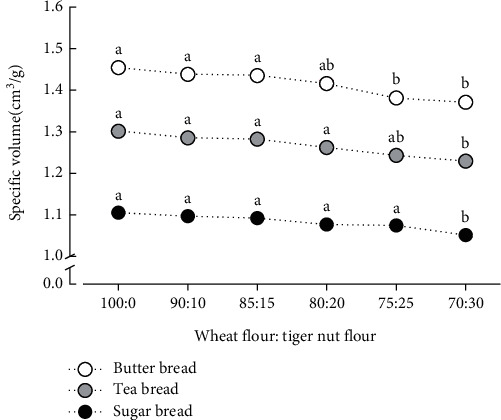
Effects of substituting wheat flour with tiger nut flour on the specific volume of butter bread, tea bread, and sugar bread. Data points marked by different letters are significantly different at *p* < 0.05.

Specific volume is an intrinsic property of bread, which immensely influences consumers' choices [24].  Figure 3 shows that the specific volume of B_b_ (1.454 ± 0.005 cm^3^/g) was the highest followed by T_b_ (1.301 ± 0.003 cm^3^/g) and S_b_ (1.105 ± 1.002 cm^3^/g), which were higher than that reported by Oke et al. [14] but lower than what was reported for T_b_ (5.74 ± 0.08 cm^3^/g) and S_b_ (4.14 ± 0.03 cm^3^/g) by Aryeetey et al. [15]. The specific volume of bread is a variable parameter, which is influenced by the type of bread, thoroughness, and duration of ingredient mixing, proofing, and baking time [25]. Substitution of bread with TNF led to a progressive reduction in specific volume (Figures 1 and 3), probably because of the reduction in gluten content, which is responsible for gas entrapment during proofing, and hence, volume increment [26]. Significant decreases in specific volume were observed after adding 20%, 25%, or 30% TNF to B_b_, T_b_, or S_b_, respectively, which was higher than that reported by Oke et al. [14], showing that the impact of TNF on the specific volume depends on the type of bread. Thus, bread type could be a major factor in determining the feasibility and suitable levels of WF substitution with TNF.

### 4.3. Effects on Crumb Hardness during Storage

**Figure 4:**
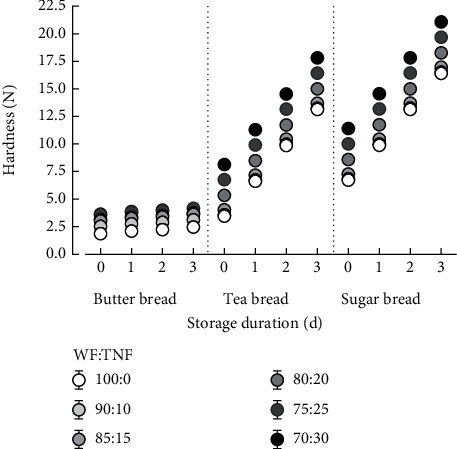
Effects of wheat flour (WF) substitution with tiger nut flour (TNF) on crumb hardness of butter bread, tea bread, and sugar bread during storage under ambient temperature (28-32°C).

Plane bread showed different levels of crumb hardness, which varied according to bread type and storage duration (Figure 4). Hardness of B_b_ (1.87 ± 0.01 N), T_b_ (3.46 ± 0.0 2 N), and S_b_ (6.71 ± 0.02 N) significantly increased to 2.46 ± 0.01 N, 13.12 ± 0.02 N, and 16.40 ± 0.02 N, respectively, after 3 d storage. Although differences in bread types exist, a similar range of bread hardness and storage-related increases were reported by Oke et al. [14], purportedly caused by moisture loss, starch retrogradation, and formation of gluten-starch crosslinks, which characterize bread staling [27]. Substitution of WF with 30% TNF caused bread hardness to increase by approx. 1.96-fold for B_b_, 2.36-fold for T_b,_ and 1.70-fold for S_b_, showing that TNF contributes to bread texture differently. Furthermore, whilst storing T_30_B_b_ for 3 d did not significantly affect the crumb hardness (4.23 ± 0.01 N), that of T_30_T_b_ and T_30_S_b_ significantly increased to 17.80 ± 0.02 N and 21.08 ± 0.02 N, respectively. This shows that although TNF increases bread hardness as reported by Oke et al. [14], the effect is dependent on the bread type, spanning from insignificant to considerable effects. Substitution of WF with TNF, which is rich in insoluble fiber [28], probably contributes to the decrease of the water binding capacity [29] and imparts rigidity/firmness to breadcrumb, resulting in higher storage-related crumb hardness. According to Gutierrez-Castillo et al. [30], interactions between fiber, protein, and gluten strengthen the gluten network, which can additionally contribute to breadcrumb compactness and hardness. The higher content of bread shortening (margarine) is known to cause dough softening because of the lipid composition [31], thus, controlling the hardness of the crumb during storage as was observed for B_b_ and its TNF substituted analogs. Bread hardness is relevant as it affects consumers' perception of anticipated chewing and judgment and strongly influences product acceptability [14].

### 4.4. Effects on Sensory Properties

**Figure 5:**
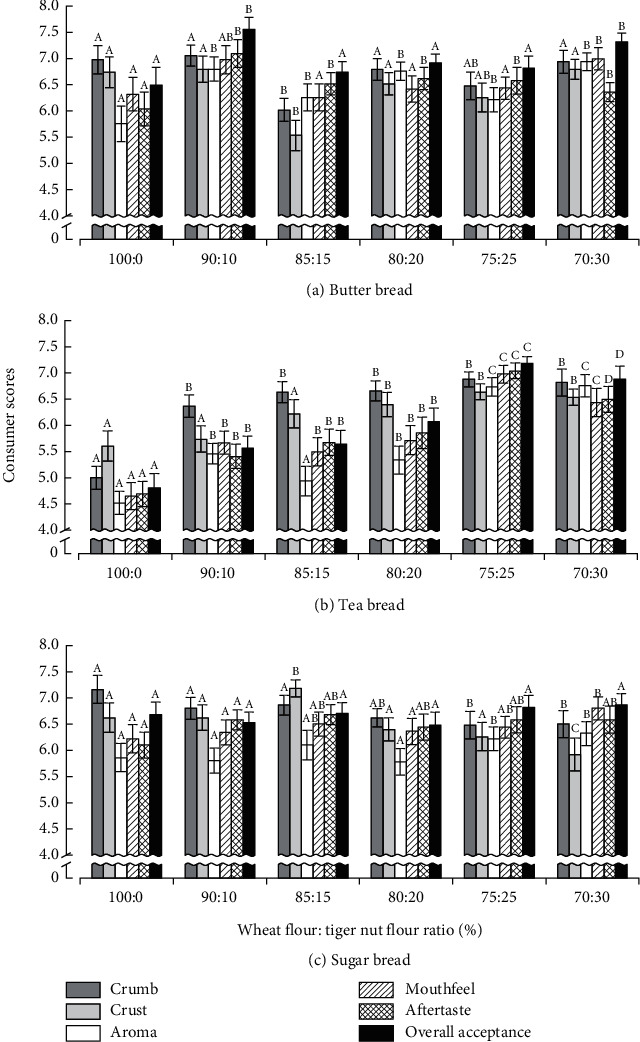
Effects of substituting wheat flour with tiger nut flour on the consumer scores for butter bread, tea bread, and sugar bread. Bars with similar colors marked with different letters are significantly different at *p* < 0.05, *N* = 56.

The impact of substituting WF with TNF on the sensory scores for bread is shown in Figure 5. Partial substitution of WF with TNF resulted in positive consumer scores for the investigated types of bread but to different extents. For example, B_b_ showed reasonably high scores for all attributes with an overall acceptance rating of 6.50 ± 0.33 (Figure 5(a)). Nonetheless, the substitution of the bread with TNF at all the levels still resulted in significantly higher scores for aroma, showing that TNF positively impacts the aroma of B_b_. Furthermore, at 10% or 30% TNF substitution, significantly higher ratings for crust appearance, mouthfeel, and overall acceptance were recorded for B_b_ with the 10% TNF additionally contributing superior scores for crumb appearance and aftertaste and resulting in the highest rating (7.56 ± 0.23) of the overall acceptance.

Tea bread (T_b_) had low consumer scores for all attributes with an overall acceptance of 4.81 ± 0.27 (Figure 5(b)). This is probably influenced by the generally low panel preference for T_b_. TNF substitution of T_b_ at all the levels investigated resulted in significantly higher scores for all attributes, except substitution at 10% and 15% TNF, which showed no significant increase in crust appearance and aroma, respectively. Substituting WF with 25% TNF resulted in the highest scores for all attributes of T_b_, increasing the overall acceptability to 7.18 ± 0.14. Probably, TNF could serve as a sensory-enhancing ingredient for T_b_, which can improve consumer acceptability, and hence, help integrate tiger nut into bakery products.

Consumer evaluation showed a high rating of all attributes of S_b_ with an overall acceptance of 6.68 ± 0.24 (Figure 5(c)). Probably, consumers had a high preference for S_b_ because of the sugar content. Mixed effects of TNF substitution of S_b_ on consumer scores were observed: substitution with 15% TNF caused a significant increase in the scores for crust appearance and aftertaste. Using 25% TNF significantly increased the scores for aroma whilst at 30% TNF substitution, higher scores than the control for aroma and mouthfeel were observed. All other substitution levels using TNF showed no significant effects on S_b_. On the contrary, scores significantly decreased for crumb and crust appearance after 30% TNF substitution, but this level showed the highest score for overall acceptability of 6.86 ± 0.22, although not significantly different from S_b_. The results show that even though some attributes of S_b_ improved by incorporating TNF, the influence on consumers' acceptability was only marginal. Although higher levels of TNF substitution were carried out in our study, the observations from the effects of S_b_ on consumer acceptance were similar to that reported by Oke et al. [14], who showed that substituting wheat bread with up to 8% TNF did not cause significant effects on consumer acceptability. On the contrary, the substitution of wheat bread with up to 10% TNF was reported to adversely affect crust color, crumb texture, aroma, and taste scores [14].

Tiger nut flour has considerable sugar content ranging from 11.54°Brix-40.38°Brix depending on the nut drying conditions [4] and is rich in fiber and oils (Table 2). Additionally, the flour shows brown characteristics with a distinct nutty or almond aroma, known to be contributed by Maillard reactions and caramelization of the sugars during the drying of the tuberous rhizomes [16]. Thus, progressive increments of TNF content in bread intensify consumer perception of TNF properties, which could affect consumer scores depending on the level of compatibility with the bread attributes.

In summary, the substitution of WF with TNF led to different consumer scores of the attributes and acceptance, which could be influenced by the nutrient composition of TNF and the type of bread. According to the results, partial substitution of WF with TNF showed an important influence on T_b_. However, because T_10_B_b_ and T_30_B_b_ revealed the highest rating for consumer acceptance, B_b_ was chosen to further investigate the effects of TNF substitution on the attributes.

**Figure 6:**
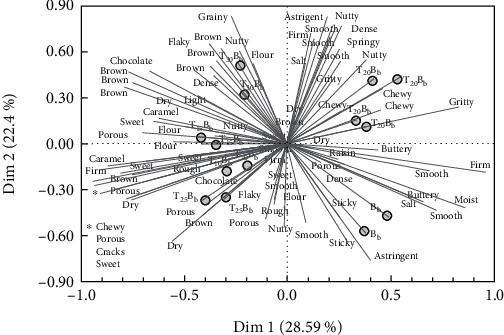
Generalized Procrustes Analyses of group average plots for descriptors of butter bread substituted with tiger nut flour. In the consensus space that is B_b_ (Butter bread), partial substitution of wheat flour with 10% tiger nut flour (T_10_B_b_), 15% tiger nut flour (T_15_B_b_), 20% tiger nut flour (T_20_B_b_), 25% tiger nut flour (T_25_B_b_), or 30% tiger nut flour (T_30_B_b_). Plots are based on duplicate experiments.

Generalized Procrustes Analyses of group average plots for descriptors of B_b_ substituted with TNF are shown in Figure 6. Principal component analysis of the consensus matrix showed that dimension 1 and dimension 2 accounted for 50.99% of the total variance and dimension 3 for 13.25%, showing a good account of data variability. The clustering of the identical coordinates of T_20_B_b_ in Figure 6 indicates that panelists were able to reliably distinguish among the samples [32]. Emerging descriptors out of the 26 different descriptors generated for B_b_ were *firm*, *moist*, *buttery*, *smooth*, and *astringent*. Those for T_10_B_b_ were *chewy*, *firm*, *sweet*, *porous*, *dry*, and *caramel* whilst T_15_B_b_ were *porous*, nutty, sweet, dry, and caramel. The most frequently used descriptors for T_20_B_b_ were *chewy*, *dry*, *springy*, nutty, gritty, and dense. Emerging descriptors for T_25_B_b_ were *dry*, nutty, flaky, brown, porous, and chewy, and that of T_30_B_b_ were *grainy*, *chocolate*, *brown*, *nutty*, *flaky*, and *grainy.* The commonly used descriptors such as *chewy and grainy*, *sweet and brown*, *flaky*, *and nutty* that characterized T_b_ substituted with TNF could be ascribed to the high tiger nut fiber content, which is known to impact bulk and cause batter cohesiveness [33]. Tiger nut sugars might have contributed to the sweet, caramel aroma of dough [34], and the oils improved the shortening and nutty properties [31]. The data shows that incorporating TNF in B_b_ affects bread attributes, which can influence consumer acceptability. The unique sensory attributes of bread generated by partially substituting WF with TNF can be exploited for producing several varieties of high-fiber bread.

### 4.5. Effects on Nutrient Composition

Nutrient composition of B_b_, T_b,_ and S_b_ and their TNF-substituted counterparts that showed the highest consumer acceptance, namely, WF substitution with 10% TNF for B_b_ (T_10_B_b_), 25% TNF for T_b_ (T_25_T_b_), and 30% TNF for S_b_ (T_30_S_b_), are shown in Table 2. The moisture content of plane bread was for B_b_, 21.09 ± 0.13%, T_b_, 26.02 ± 0.03%, and S_b_, 26.70 ± 0.39%. The moisture values were comparable to that reported by Oke et al. [14], although the recipes differ. Contrary to the reported decrease in moisture of TNF-substituted bread by Oke et al. [14], this study showed no significant effects on moisture content, which were for T_10_B_b_, 21.83 ± 0.07%; T_25_T_b_, 26.87 ± 0.4%, and T_30_S_b_, 25.73 ± 0.04%. The low moisture of B_b_ and T_10_B_b_ may be attributed to the high margarine content, which is known to decrease water binding because of the coating effects, rendering a higher propensity to bake-related moisture loss [34]. Low moisture may have relevance for improved shelf life [35].

The nutrient composition of the bread was within the range reported by Maietti et al. [36] and Oke et al. [14]. Substitution of bread using TNF significantly increased the content of fat, total fiber, and ash and caused the protein and carbohydrate content to decrease. Apart from the decrease in carbohydrate, the effects of adding TNF to bread on the nutrient composition followed a similar trend as reported by Oke et al. [14] but differed in terms of the magnitude because of the type of bread, percentage substitution, and TNF composition. The nutrient composition of WF used in this study was for protein, 14.63 ± 0.34 g/100 g; fiber, 1.45 ± 0.04 g/100 g; ash, 0.63 ± 0.01 g/100 g; fat, 4.89 ± 0.02 g/100 g; and carbohydrate, 74.40 ± 0.38 g/100 g. Thus, the substitution of WF with TNF, which had higher total fiber, fat, and ash content but lower protein (Table 2), caused corresponding variations in the nutrient content of the bread composites. Tiger nut comprises nutritionally valuable lipids (at least 65% monounsaturated fatty acids), several important minerals, dietary fiber, and bioactive phytochemicals [1, 37], which can contribute to essential physiological functions [2, 38] and improve digestion [39]. Thus, the substitution of WF with TNF could create healthy bread alternatives for consumers. The inclusion of a larger consumer population for the sensory study could strengthen the conclusions regarding bread acceptability.

## 5. Conclusion

The feasibility of tiger nut utilization for bread processing was experimented by partially substituting wheat flour (WF) with tiger nut flour (TNF) at a ratio of 100 : 0, 90 : 10, 85 : 15, 80 : 20, 75 : 25, and 70 : 30 for butter bread (B_b_), tea bread (T_b_), and sugar bread (S_b_) to determine the bread type that is more amenable to tiger nut incorporation. Generally, substituting WF with TNF increased the brownness and color saturation, decreased bread lightness with a greater influence on the crust than the crumb, and showed the greatest impact on S_b_, followed by T_b_ and B_b_. The incorporation of TNF in bread progressively decreased the specific volume. The amount of TNF that was required to significantly impact specific bread volume was the highest for S_b_ followed by T_b_ and B_b_. Furthermore, bread hardness increased with TNF substitution, showing the greatest impacts on T_b_, followed by B_b_ and S_b_. Although ambient storage further increased bread crumb hardness of T_b_ and S_b,_ that of B_b_ did not significantly change. Regarding consumer evaluation, substituting B_b_ with 10%TNF gave the highest overall acceptance. Consumer scores for T_b_ were generally low; however, adding TNF significantly improved the scores for all attributes, and substitution using 25% TNF gave the highest scores for overall acceptance. Descriptors for B_b_ improved after substituting WF with TNF. Thus, WF substitution with TNF showed that B_b_ with 10% or T_b_ with 25% TNF is more comparable with the overall acceptance quality of 100% WF. The results are relevant for enhancing the utility of tiger nuts as an ingredient in bread processing to generate fiber-rich, palatable, and probably, more affordable bread alternatives. Future studies would assess the cost of tiger nut bread production and willingness-to-pay among bread consumers.

## Figures and Tables

**Table 1:** Recipe for tea bread, sugar bread, and butter bread [15].

Ingredients (g)	Tea bread	Sugar bread	Butter bread
Flour	1250	1250	1250
Margarine	144	144	288
Salt	9	6	9
Sugar	68	214.5	68
Yeast	0.6	0.6	0.6
Nutmeg	3	3	3
Water (mL)	650	650	650

**Table 2:** Effects of substituting wheat flour with tiger nut flour on butter bread, tea bread, and sugar bread on the nutrient composition (g/100 g).

Nutrient compound	Tiger nut flour	^∗^Butter bread		Tea bread		Sugar bread	
		Without tiger nut flour	With 10% tiger nut flour	Without tiger nut flour	With 25% tiger nut flour	Without tiger nut flour	With 30% tiger nut flour
Protein	6.55 ± 0.23	12.67 ± 0.35^a^	11.86 ± 0.33^b^	13.56 ± 0.28^a^	11.14 ± 0.34^b^	12.92 ± 0.09^a^	10.90 ± 0.08^b^
Fat	8.25 ± 0.06	15.29 ± 0.07^a^	16.30 ± 0.08^b^	9.03 ± 0.01^a^	10.04 ± 0.01^b^	10.50 ± 0.22^a^	11.34 ± 0.22^b^
Total fiber	9.52 ± 0.11	1.29 ± 0.05^a^	2.10 ± 0.04^b^	1.07 ± 0.04^a^	3.49 ± 0.01^b^	3.15 ± 0.09^a^	5.17 ± 0.06^b^
Ash	2.59 ± 0.05	1.43 ± 0.04^a^	1.62 ± 0.04^b^	1.30 ± 0.06^a^	1.89 ± 0.07^b^	1.06 ± 0.02^a^	1.56 ± 0.02^a^
Carbohydrate	73.09 ± 0.30	69.32 ± 0.48^a^	68.79 ± 0.46^b^	75.04 ± 0.25^a^	73.45 ± 0.29^b^	72.36 ± 0.17^a^	71.04 ± 0.15^a^

^∗^Values in the same rows of bread categories marked by different superscripts are significantly different at *p* < 0.05.

## Data Availability

Data supporting the findings of the study are available within the manuscript.

## Abbreviations

WF: Hard wheat flour
B_b_: 100% wheat flour butter bread
T_b_: 100% wheat flour tea bread
S_b_: 100% wheat flour sugar bread
TNF: Tiger nut flour
T_10_B_b_: Butter bread substituted with 10% tiger nut flour
T_15_B_b_: Butter bread substituted with 15% tiger nut flour
T_20_B_b_: Butter bread substituted with 20% tiger nut flour
T_25_B_b_: Butter bread substituted with 25% tiger nut flour
T_30_B_b_: Butter bread substituted with 30% tiger nut flour
T_25_T_b_: Tea bread substituted with 25% tiger nut flour
T_30_S_b_: Sugar bread substituted with 30% tiger nut flour.
